# Twelve-Month Follow-Up and Economic Evaluation of an Alternative Care Provider Clinic for Severe Sleep-Disordered Breathing

**DOI:** 10.1016/j.chest.2026.01.008

**Published:** 2026-01-24

**Authors:** Erika D. Penz, Ada Ip-Buting, Willis H. Tsai, Maria J. Santana, W. Ward Flemons, Kristin L. Fraser, Sachin R. Pendharkar

**Affiliations:** a Division of Respirology and Sleep Medicine, University of Saskatchewan, Saskatoon, SK, Canada; b Department of Medicine, Cumming School of Medicine, University of Calgary, Calgary, AB, Canada; c Department of Community Health Sciences, Cumming School of Medicine, University of Calgary, Calgary, AB, Canada; d Department of Paediatrics, Cumming School of Medicine, University of Calgary, Calgary, AB, Canada; e O’Brien Institute for Public Health, University of Calgary, Calgary, AB, Canada

**Keywords:** alternative care provider, cost, cost-effectiveness, quality of life, sleep-disordered breathing

## Abstract

**Background:**

Use of nonphysician alternative care providers (ACPs) can improve timely access to sleep-disordered breathing (SDB) care, and previous studies have demonstrated beneficial short-term clinical outcomes. Longer-term clinical and economic impacts of an ACP model for patients with severe SDB have not been evaluated.

**Research Question:**

Is ACP-led care of SDB effective and cost-effective compared with standard care by a sleep physician?

**Study Design and Methods:**

One-year follow-up data from a randomized controlled trial conducted among patients with severe SDB, randomized 1:1 to physician- or ACP (respiratory therapist)-led management is reported. Clinical outcomes included adherence to positive airway pressure therapy and patient-reported outcomes including sleepiness, quality of life, and care satisfaction. A cost-utility analysis was conducted using trial data and health administrative data, with costs calculated from the perspective of the Canadian public payer and quality-adjusted life years (QALYs) using Health Utility Index scores. Willingness to pay for incremental cost-effectiveness ratio estimates were summarized with cost-effectiveness acceptability curves. Referral wait times and polysomnogram use were evaluated in subgroup analyses.

**Results:**

Adherence, treatment efficacy, and patient-reported outcomes were similar among ACP-led and standard care arms. Mean utility scores improved over the 1-year period in both groups. The estimated incremental cost-effectiveness ratio associated with the ACP-led group compared with standard care was −$34,580 per QALY. The probability the ACP-led clinic was cost-effective compared with standard care was 61.9% at a willingness to pay threshold of $50,000/QALY. Subgroup and sensitivity analyses did not alter results.

**Interpretation:**

Our results show that the ACP-led management strategy demonstrated similar clinical outcomes 1 year after treatment initiation. This model is likely to be cost-effective relative to standard care among patients with severe SDB. Results were robust to sensitivity analysis and support implementation of the ACP care model in this population.

**Take-Home Points**

**Research Question:** Is alternative care provider (ACP)-led care of severe sleep-disordered breathing effective and cost-effective compared with standard care by a sleep physician?

**Results:** Adherence, treatment efficacy, patient-reported outcomes, and costs were similar among ACP-led and standard care arms.

**Interpretation:** The ACP-led management strategy demonstrated similar clinical outcomes 1 year after treatment initiation and is likely to be cost-effective relative to standard care among patients with severe sleep-disordered breathing.

Sleep-disordered breathing (SDB) encompasses several conditions including OSA, central sleep apnea, and sleep-related hypoventilation.^1^ OSA is the most common SDB and is estimated to affect nearly 1 billion adults worldwide.^2^ There are serious consequences of untreated OSA, including an increased risk of hypertension, cardiometabolic disease, and motor vehicle accidents.^3^, ^4^, ^5^, ^6^, ^7^ In addition to the increased risk of morbidity, mortality, and impairment on quality of life, OSA is associated with higher health care utilization and expenditures. Individuals with untreated OSA are estimated to have a 2- to 3-fold increase in medical costs compared with individuals without OSA.^8^^,^^9^ The economic burden of untreated OSA in the United States has been estimated to be $149.6 billion annually.^10^ Treatment of OSA is effective, and cost-effective, in part due to the prevention of major health complications.^3^^,^^11^, ^12^, ^13^, ^14^, ^15^, ^16^, ^17^, ^18^ Patients with sleep hypoventilation are at especially high risk of complications without adequate treatment.^19^, ^20^, ^21^, ^22^

Challenges in providing timely access to sleep specialist care are well described and may lead to underdiagnosis of SDB and compromised effectiveness of treatment; the importance of timely access is even more important for individuals with severe SDB.^23^, ^24^, ^25^, ^26^, ^27^, ^28^, ^29^, ^30^, ^31^, ^32^ Strategies to improve access and wait times, such as home sleep apnea testing (HSAT) or telemedicine, are still dependent on the supply of sleep physicians, which is inadequate in many jurisdictions.^22^^,^^33^, ^34^, ^35^, ^36^ Management of OSA by nonphysician alternative care providers (ACPs) such as nurses and respiratory therapists has been proposed to improve timely access to care. Observational studies and randomized trials have demonstrated that ACP management of uncomplicated OSA leads to comparable outcomes compared with sleep specialist care, and is potentially less costly.^37^, ^38^, ^39^ However, the application of such models to those with more severe or medically complex SDB, for whom the consequences of delays may be more pronounced, has not been well studied.

We previously conducted a randomized controlled noninferiority trial comparing an ACP clinic with physician-led care.^40^ At 3 months, we found that ACP care was indeterminate for noninferiority with respect to positive airway pressure (PAP) adherence but had greater benefit on patient-reported outcomes. In this paper, we report clinical and patient-reported outcomes from the trial after 12 months of follow-up, and a cost-utility analysis of the ACP clinic compared with physician-led management (standard care) using costs and health-related quality of life data collected during the clinical trial. We hypothesized that the ACP clinic would lead to similar clinical outcomes and be cost-effective compared with standard, physician-led care.

## Study Design and Methods

### Study Design

This study reports 1-year follow-up data from a randomized controlled trial.^40^ An economic evaluation (cost-utility analysis) was conducted using data collected during the study and supplemented with electronic health administrative data, from the perspective of the public payer. Quality-of-life outcomes and health care-related use and costs were calculated using a 1-year time horizon, from initial assessment in clinic to 1 year after treatment initiation. No discounting rate was applied to costs or outcomes. The study was approved by the University of Calgary Conjoint Health Research Ethics Board (REB13-1280) and registered on clinicaltrials.gov (NCT02191-085).

### Study Population and Procedure

A detailed protocol of the randomized controlled trial has been published previously.^41^ Briefly, the study was conducted at the Foothills Medical Centre (FMC) Sleep Centre, a publicly funded, tertiary academic sleep center in Calgary, Alberta, Canada. The FMC Sleep Centre receives approximately 2,500 referrals annually, 30% of which are for severe SDB (defined as severe OSA with or without hypoventilation). Patients were eligible for inclusion in the study if there was strong clinical suspicion of SDB at time of referral, which was determined by meeting at least 1 of the inclusion criteria: severe OSA (Oxygen Desaturation Index [ODI] ≥ 30 events/h on type 3 HSAT, mean nocturnal oxygen saturation ≤ 85% on HSAT) or suspected sleep hypoventilation syndrome (ODI ≥ 15 events/h on HSAT and Pco_2_ ≥ 45 mm Hg on arterial blood gas). Patients were excluded if they had a suspected concomitant sleep disorder other than SDB, had been previously treated with PAP therapy for SDB, had health insurance outside of Alberta, or did not consent to participate in the study.

Eligible patients, who were referred to the FMC Sleep Centre for severe SDB between October 2014 and May 2017 and consented to participate, were randomized 1:1 to standard physician-led SDB management or an ACP-led clinic in which SDB management was provided by 1 of 5 ACPs under the supervision of 1 of 6 sleep physicians. ACPs were respiratory therapists who were registered in the province of Alberta and had at least 5 years’ experience managing patients with SDB. Depending on the treatment arm, a sleep physician or ACP performed the initial assessment and developed the management plan. In the ACP arm, a sleep physician reviewed the management plan in real time and briefly met the patient to address additional health, sleep-related, or safety concerns before confirming the plan for further tests and/or treatment. In both groups, follow-up appointments were delegated to the ACP if deemed appropriate by the initial provider, and ACPs could refer patients back to the primary sleep physician at any point for clinical issues outside of the respiratory therapists’ scope of practice. Follow-up and indications for requesting a physician appointment were guided by established physician-approved protocols at the FMC Sleep Centre.

Demographic and clinical data were obtained from the FMC Sleep Centre electronic medical record. At 3 and 12 months after treatment initiation, we obtained repeat HSAT on treatment and PAP usage data from machine downloads and patient-reported outcome data using paper questionnaires. Questionnaires included the Epworth Sleepiness Scale, Health Utilities Index Mark 3 (HUI3), short-form Sleep Apnea Quality of Life Index, and Visit-Specific Satisfaction Instrument.^42^, ^43^, ^44^, ^45^, ^46^ Clinic visit duration at the FMC Sleep Centre was collected to determine time allocated by sleep physicians and ACPs performing clinical assessment and documentation, which was used to inform cost estimates. Electronic administrative health data were obtained from Alberta Health Services Analytics and included outpatient pharmacy dispensing records, hospitalizations, emergency department visits, and physician billing records. Data were accessed through Data and Analytics of Alberta Health Services.

Quality of life was measured using the HUI3, a self-administered questionnaire examining 8 attributes including vision, hearing, speech, ambulation, dexterity, emotion, cognition, and pain, over a range of 5 to 6 levels.^42^ Patient responses to the HUI3 questionnaire were converted to a utility score ranging from 0 (death) to 1.0 (perfect health). Quality-adjusted life-years (QALYs) were estimated by combining the total length of follow-up time and utility scores for each patient at baseline and 3 and 12 months, using area under the curve methodology.^47^ Given that the baseline utility scores were different between the 2 groups, we adjusted for this in our analysis using the change from baseline-area under the curve methodology.^48^

Health care resource utilization was taken from clinical trial data and the electronic health administrative data on drug dispensations, physician services (fee-for-service codes), hospitalization, and emergency department visits. Nonmedical costs (ie, patient time and travel costs, costs related to lost productivity) were not included. Drug prices were taken from the Drug Products and Pricing Alberta Drug Benefit List database and linked to the pharmacy data to calculate the drug costs for each dispensation. Hospitalization and emergency department costs were calculated using a cost-weighted case approach, with Alberta-specific hospitalization cost weights. Physician costs were based on fee-for-service claims taken from the practitioner claims data set, and sleep clinic-specific costs (including ACP visits) and equipment were based on internal microcosting conducted at the clinic (Table 1). Costs were calculated from the perspective of the public health care payer and converted to 2019 Canadian dollars using the health care component of the Canadian Consumer Price Index.^49^

**Table 1:** Data Sources and Costs

Variables	Data Sources	Notes
Patient identifier	Provincial health number	Identifiers were scrambled and deidentified
Patient characteristics		
Patient demographics	FMC Sleep Centre electronic medical record	Collected as part of the clinical trial
Sleep testing data	FMC Sleep Centre electronic medical record	
Comorbidities/medications	Patient self-report	
PAP usage	Machine downloads	
Patient-reported outcomes	Questionnaires; ESS, HUI2, HUI3, SAQLI, VSQ-9 (12 mo)	
Health care utilization and costs		
Hospitalizations	CIHI DAD	Costs were calculated using CIHI Resource Intensity Weight methodology
Emergency department visits	CIHI NACRS	
Sleep clinic costs	Case report forms	Costs were calculated based on microcosting exercise
Outpatient drug dispensations	AHS - Pharmaceutical Information Network	Costs were calculated using Alberta Drug Benefit List costs
Physician services	AHS - Practitioner Claims database	Costs based on fee paid per claim

AHS = Alberta Health Services; CIHI = Canadian Institutes for Health Information; DAD = Discharge Abstract Database; ESS = Epworth Sleepiness Scale; FMC = Foothills Medical Centre; HUI2 = Health Utilities Index Mark 2; HUI3 = Health Utilities Index Mark 3; NACRS = National Ambulatory Care Reporting System; PAP = positive airway pressure; SAQLI = Sleep Apnea Quality of Life Index; VSQ-9 = Visit-Specific Satisfaction Instrument.

### Analysis

In the primary analysis, we evaluated PAP adherence in the 4 weeks before the 3-month treatment follow-up for all patients in the primary analysis (n = 156).^40^ Adherence was reported as average nightly PAP use and dichotomized based on a threshold of 4 hours of use per night for at least 70% of nights.^50^ We also reported wait times for initial assessment and treatment and several patient-reported outcomes including sleepiness, general and disease-specific health-related quality of life, and patient satisfaction. In the present study, the main outcomes included PAP use (measured in the 4 weeks before the 12-month time point), patient-reported outcomes at 1 year, and direct health care utilization and costs. A modified intention-to-treat analysis was used that included patients who were randomized and had at least 1 visit to the FMC Sleep Centre. If objective adherence data were not available at 12 months, participants were assumed to have zero adherence. Paired *t* tests were used to compare clinical outcomes, and unpaired *t* tests were used to compare system outcomes. Proportions were assessed using χ^2^ tests. The sample size of 128 patients in the primary study was determined based on a noninferiority margin for difference in PAP adherence of −1 hour at 3 months, and provided power of 0.9 with a type I error of 0.05 assuming an SD for adherence of 2 hours.^41^

Incremental cost-effectiveness ratio (ICER) was calculated as the difference in costs between the ACP-led and standard care groups divided by the difference in QALYs gained between the 2 groups, as in the following equation: $\text{ICER}=\frac{C_{1}-C_{0}}{E_{1}{-E}_{0}}=\frac{\Delta C}{\Delta E}$, where C1 (E1) and C0 (E0) are the mean costs (QALYs) in the ACP-led clinic and standard care groups, respectively, and ΔC and ΔE are the difference in mean costs and mean QALYs between the 2 groups. Differences in QALYs were calculated after adjusting for difference in baseline utility scores. ICER represents the incremental costs associated with an additional QALY gained from the intervention (ie, ACP-led clinic), and is reported as dollars spent per QALY gained. Analysis was completed using an intention to treat protocol. We used nonparametric bootstrapping with 1,000 replications to derive a 95% CI for the incremental mean cost difference and mean QALY difference between the groups. For the bootstrap estimate, we used the percentile method.^51^ Difference in mean costs and mean QALYs for each of the 1,000 data sets was calculated, ranked from lowest to highest, and the difference in mean cost and QALY for the 26th and 975th ordered values defined the 95% CI. The uncertainty around the ICER estimates was summarized using cost-effectiveness acceptability curves, reporting the probability the ACP-led clinic is cost-effective compared with standard care at a willingness to pay (WTP) threshold value of $0/QALY and $50,000/QALY.^52^

As a secondary analysis, we reported cost-effectiveness using the net monetary benefit (NMB) regression framework methodology.^53^^,^^54^ The NMB measures the NMB (in dollars) of the ACP-led clinic vs standard care, for a given WTP threshold for a QALY gain; NMB values > 0 indicate the intervention is cost-effective at a given WTP threshold. This approach uses a parametric linear regression framework and allows for the incorporation of covariates to control for differences between the 2 treatment arms at baseline (ie, sex, age at baseline, baseline HUI3 utility score, number of comorbidities). We used WTP thresholds of $0 and $50,000 to evaluate differences in the NMB of the ACP-led clinic compared with standard care. All analyses were conducted in R Studio (RStudio Team).

Missing health utility data were estimated using multiple imputation methods to minimize bias in the analysis and increase precision.^55^ The imputation method used a predictive mean matching approach. We considered missing data to be missing at random.

### Base Case Analysis, Subgroup Analysis, and Sensitivity Analysis

An intention to treat analysis of the ICER between the ACP-led clinic and standard care was completed after 1 year of SDB treatment, adjusted for difference in baseline utility scores. Multiple imputation was applied for missing quality of life data. In the base case scenario, 1 patient in the standard care group had health care utilization costs > $200,000 over the follow-up period. Given these high costs, we chose to exclude this individual to reduce biased cost results in favor of the ACP-led clinic.

Subgroup analyses were conducted to identify whether differences in referral wait times (< 90 or > 90 days) and use of polysomnogram (vs HSAT alone) altered the cost-effectiveness of the ACP-led clinic compared with standard care.^33^^,^^56^

Several sensitivity analyses were conducted to confirm the robustness of the results and assumptions used in the base case scenario. We varied the unit prices of health care resources (respiratory therapists’ salaries, diagnostic tests, and sleep clinic costs), performed a complete case analysis, and included the individual with excessively high health care costs (> $200,000).^19^^,^^24^

## Results

**Figure 1:**
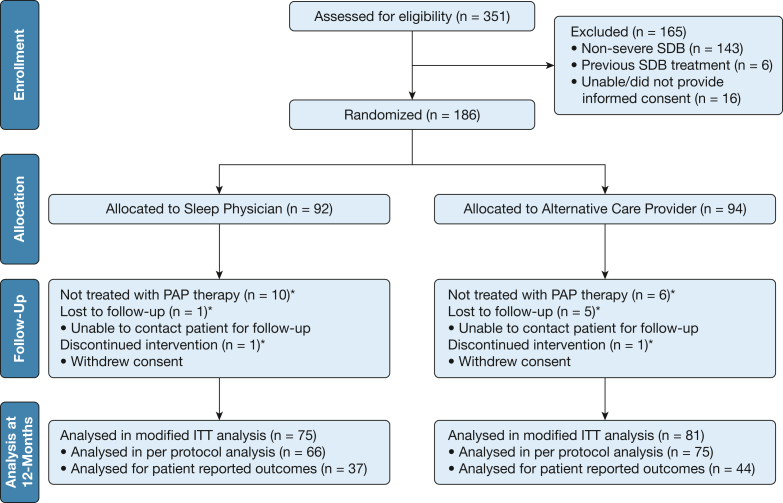
Participant flow. ∗Reasons for exclusion may not sum to 100% because some patients met multiple exclusion criteria. ITT = intention to treat; PAP = positive airway pressure; SDB = sleep-disordered breathing. Reprinted from Pendharkar et al^40^ with permission of the American Thoracic Society. Copyright © 2025 American Thoracic Society. All rights reserved. The American Journal of Respiratory and Critical Care Medicine is an official journal of the American Thoracic Society.

A total of 156 patients participated in the study, with 75 randomized to standard care and 81 randomized to the ACP-led clinic arm. At 12 months, patient-reported outcome data were available for 37 patients in the standard care group and 44 patients in the ACP group (Fig 1). Baseline characteristics were generally balanced between the treatment arms (Table 2). The overall HUI3 score at baseline was slightly lower in the standard care group (0.56) compared with the ACP group (0.59), but this finding was not statistically significant (*P* = .49).

**Table 2:** Baseline Characteristics

Characteristic	Standard Care		ACP-Led Clinic	
	(n = 75)		(n = 81)	
Male sex	54	(72)	58	(72)
Age at recruitment, y	55	[13]	54	[12]
BMI, kg/m^2^	39	[9]	40	[10]
Comorbidities				
Hypertension	47	(63)	49	(60)
Diabetes	21	(28)	21	(26)
Cardiovascular disease	16	(21)	15	(19)
Chronic lung disease	16	(21)	23	(28)
Chronic kidney disease	5	(7)	0	(0)
ODI, events/h	55	[29]	51	[28]
HSAT mean Spo_2_, %	86	[5]	85	[5]
AHI, events/h^a^	77	[50]	75	[59]
PSG mean Spo_2_, %^a^	86	[5]	85	[5]
Treatment type, n (%)^b^				
CPAP	59	(79)	64	(79)
BPAP	6	(8)	11	(14)
Oxygen	8	(11)	11	(14)
No therapy	6	(8)	4	(5)
ESS score	11	[6]	11	[5]
Overall HUI3	0.56	[0.32]	0.59	[0.31]
SAQLI	4.6	[1.3]	4.5	[1.4]

Data are presented as No. (%) or mean [SD] or as otherwise indicated. ACP = alternative care provider; AHI = apnea-hypopnea index on polysomnography; BPAP = bilevel positive airway pressure; ESS = Epworth Sleepiness Scale; HSAT = home sleep apnea testing; HUI3 = Health Utilities Index Mark 3; PSG = polysomnography; ODI = Oxygen Desaturation Index; SAQLI = Sleep Apnea Quality of Life Index; Spo_2_ = oxygen saturation by pulse oximetry.

a Data presented only for patients who underwent PSG (46 for sleep physician arm, 40 for alternative care provider arm).

b Treatment type may sum to > 100% because some patients used oxygen in addition to CPAP or BPAP.

Adherence and secondary outcomes at 1 year are reported in Tables 3 and 4, respectively. No difference was observed in adherence defined as a continuous or categorical variable. Treatment efficacy and patient-reported outcomes were also similar.

**Table 3:** PAP Adherence at 1 Year

Variables	Standard Care (n = 75)	ACP-Led Clinic (n = 81)	Difference	*P* Value
PAP use on all nights, h/night	2.74 (1.99-3.49)	2.97 (2.21-3.73)	0.23 (−0.83 to 1.28)	.67
PAP use on nights use, h/night	3.09 (2.3-3.89)	3.2 (2.42-3.98)	0.11 (−1 to 1.21)	.85
Adherence, %^a^	0.35 (0.24-0.46)	0.41 (0.3-0.52)	0.06 (−0.11 to 0.24)	.54

Data are presented as mean (95% CI) or as otherwise indicated. ACP = alternative care provider; PAP = positive airway pressure.

a If PAP download revealed at least 4 h of PAP use on at least 70% of nights in the prior month. 37 participants in the standard care arm and 38 participants in the ACP arm had objective adherence data at the 12-mo time point; for participants with missing adherence data, adherence was assumed to be zero.

**Table 4:** Secondary Outcomes at 1 Year

Variables	Standard Care				Alternative Care Provider				Difference	
	No.	Baseline Mean (95% CI)	1-y Mean (95% CI)	Change Mean (95% CI)	No.	Baseline Mean (95% CI)	1-y Mean (95% CI)	Change Mean (95% CI)	Difference	*P* Value
ESS score	37	10.03 (8.29 to 11.76)	5.41 (3.91 to 6.9)	−4.62 (−6.88 to −2.37)	44	11.48 (9.77 to 13.19)	5.07 (3.87 to 6.26)	−6.41 (−8.07 to −4.75)	−1.79 (−4.55 to 0.97)	.20
ODI events/h	33	56.83 (45.58 to 68.08)	11.11 (5.25 to 16.98)	−45.72 (−58.01 to −33.43)	32	50.38 (39.23 to 61.54)	6.85 (3.51 to 10.19)	−43.53 (−55.53 to −31.54)	2.18 (−14.66 to 19.02)	.80
HUI3	37	0.56 (0.44 to 0.66)	0.7 (0.6 to 0.8)	0.15 (0.05 to 0.24)	44	0.57 (0.48 to 0.67)	0.72 (0.63 to 0.81)	0.15 (0.06 to 0.23)	0 (−0.13 to 0.12)	.99
SAQLI	37	4.59 (4.17 to 5.01)	5.58 (5.14 to 6.02)	0.99 (0.45 to 1.53)	44	4.4 (4 to 4.81)	5.79 (5.45 to 6.14)	1.39 (1 to 1.78)	0.4 (−0.26 to 1.06)	.23
VSQ-9	35	NA	34.54 (31.74 to 37.34)	NA	44	NA	37.39 (35.54 to 39.23)	NA	1.49 (−1.93 to 4.91)	.39

ESS = Epworth Sleepiness Scale; HUI3 = Health Utilities Index Mark 3; NA = not applicable; ODI = Oxygen Desaturation Index; SAQLI = Sleep Apnea Quality of Life Index; VSQ-9 = Visit-Specific Satisfaction Instrument.

### Utility Scores

Mean utility scores ± SD improved over the study period for both groups (Table 5). Mean bootstrapped QALYs were 0.654 ± 0.289 and 0.587 ± 0.306 in the ACP-led and standard care groups, respectively, for a mean QALY difference of 0.068 (95% CI, −0.03 to 0.167) in the unadjusted analysis. When adjusting for difference in baseline utility scores, the mean QALY difference between the ACP-led and standard care group was lower in magnitude and was not statistically significant (0.01; 95% CI, −0.03 to 0.05).

**Table 5:** Costs, Quality of Life, and Economic Outcomes for Base Case

Variables	Standard Care (n = 75)	ACP (n = 80)	Difference (ACP-Standard Care)^a^	
Summary of Cost Components ($CAD)	Mean [SD]	Mean [SD]	Mean	95% CI
Physician costs (fee-for-service)	1,960 [2,440]	1,670 [2,430]	−305	−1090 to 522
Outpatient drug costs	1,780 [2,640]	1,360 [2,040]	−419	−1201 to 317
Hospital services	1,410 [4,410]	1,760 [5,930]	320	−1,334 to 2,018
Emergency department visits	282 [831]	219 [585]	−61	−315 to 167
Sleep clinic costs	3,110 [1480]	3,190 [1720]	97	−388 to 590
Total costs	8,530 [7,740]	8,210 [9,210]	−369	−2,946 to 2,370
Health-related quality of life measure				
Overall HUI3 utility score at baseline	0.56 [0.32]	0.59 [0.32]	0.035	−0.06 to 0.14
Overall HUI3 utility score at 3 mo	0.65 [0.32]	0.73 [0.28]	0.073	−0.03 to 0.18
Overall HUI3 utility score at 1 y	0.7 [0.32]	0.72 [0.30]	0.02	−0.12 to 0.16
Bootstrapped costs and QALYs				
Total costs	8,572 [7,617]	8,203 [8,841]	−369	−2,946 to 2,370
QALYs	0.587 [0.306]	0.654 [0.289]	0.068	−0.03 to 0.167
QALYs (change from baseline)^b^	0.06	0.07	0.01	−0.03 to 0.05

Cost-Effectiveness Measures	Value	95% CI	*P* Value
Incremental cost-effectiveness ratio	−34,580	NA	NA
Likelihood of cost-effectiveness at WTP = $0/QALY	43.3%	NA	NA
Likelihood of cost-effectiveness at WTP = $50,000/QALY	61.9%	NA	NA
Net monetary benefit at WTP = $0/QALY^c^	$413	−$2,135 to $2,962	.75
Net monetary benefit at WTP = $50,000/QALY^c^	$1,050	−$2,225 to $4,325	.53

ACP = alternative care provider; HUI3 = Health Utilities Index Mark 3; NA = not applicable; QALY = quality-adjusted life-year; WTP = willingness to pay.

a Mean cost differences are bootstrapped.

b Adjusted with patient baseline characteristics.

c Adjusted for differences at baseline (ie, sex, age at baseline, baseline HUI3 utility score, number of comorbidities).

### Costs

**Figure 2:**
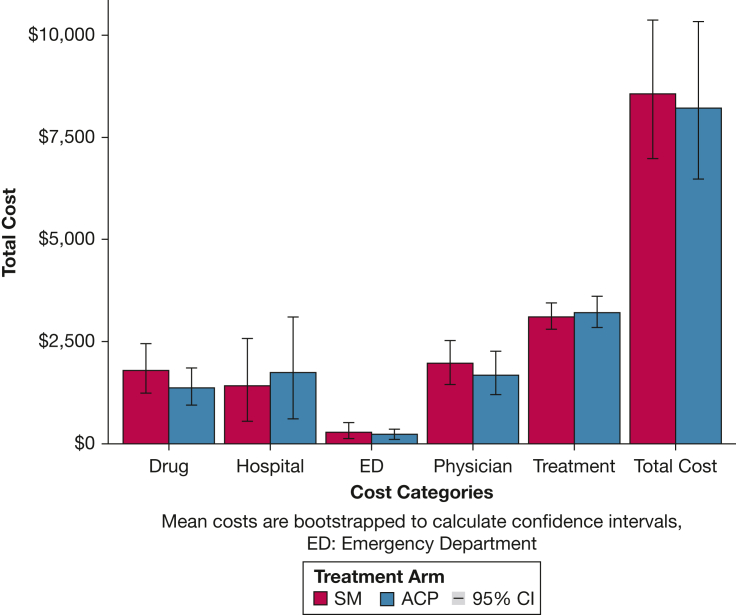
Mean health care costs by treatment group. Mean costs are bootstrapped to calculate CIs. Costs are in Canadian dollars. ACP = alternative care provider; ED = emergency department; SM = standard management.

Mean total health care costs ± SD were $8,203 ± $8,841 and $8,572 ± $7,617 for the ACP-led and standard care groups, respectively, over the follow-up period. Total mean costs were lower for the ACP-led group compared with standard care; however, the difference was not statistically significant (−$369; 95% CI, −2,946 to 2,370). Breakdown of costs are presented in Table 5 and Figure 2; across all the cost categories, the mean costs were similar between groups.

### Economic Evaluation

**Figure 3:**
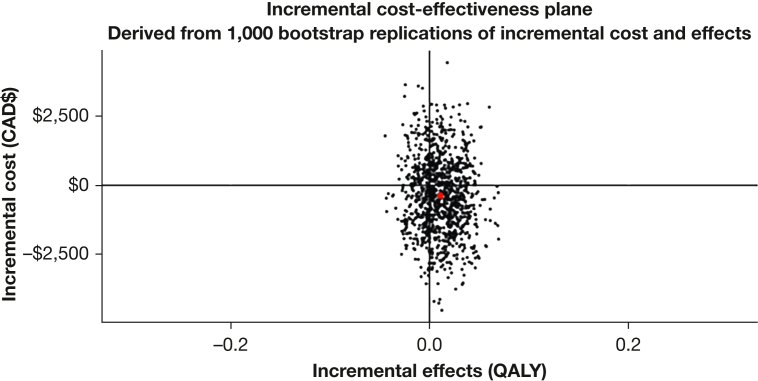
Cost-effectiveness plane for base case. Incremental cost-effectiveness plane, derived from 1,000 bootstrap replications of incremental costs and effects. QALY = quality-adjusted life year.

**Figure 4:**
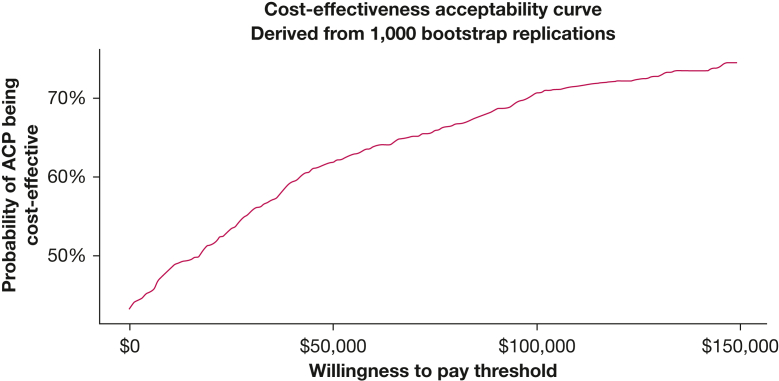
Cost-effectiveness acceptability curves for base case. Cost-effectiveness acceptability curve, derived from 1,000 bootstrap replications. Willingness to pay thresholds in Canadian dollars. ACP = alternative care provider.

In the base case analysis, the incremental QALY difference between ACP-led and standard care groups was 0.01 (95% CI, −0.03 to 0.05), and incremental costs were −$369 (95% CI, −2,946 to −2,370). After bootstrapping, the estimated ICER associated with the ACP-led group compared with standard care was −$34,580 per QALY gained. The cost-effectiveness plane, shown in Figure 3, plots the 1,000 bootstrap ICERs, which are primarily located in the bottom-right (dominant) quadrant of the graph, indicating the ACP-led clinic compared with standard care is most often less costly and associated with additional quality of life gains. The cost-effectiveness acceptability curves (Fig 4) demonstrated that the probability the ACP-led clinic was cost-effective compared with standard care was 61.9% at a WTP threshold of $50,000/QALY and 43.3% at a threshold of $0/QALY (ie, not willing to spend additional dollars to achieve additional health gains).

### Subgroup and Sensitivity Analysis

**Supplementary Figure 1:**
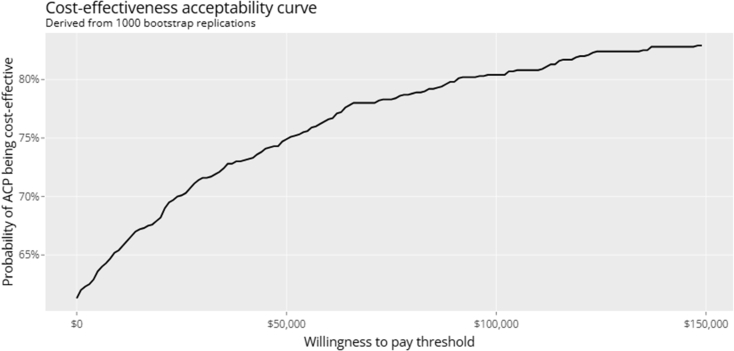
Cost-effectiveness acceptability curves for complete cases. Cost-effectiveness acceptability curve, derived from 1,000 bootstrap replications. Willingness to pay thresholds in Canadian dollars. ACP = alternative care provider.

Results of subgroup and sensitivity analyses are presented in Table 6 and e-Figure 1. Overall, subgroup and sensitivity analyses did not alter the cost-effectiveness analysis results.

**Table 6:** Summary Results of Economic Outcomes From Sensitivity and Subgroup Analysis

Scenario	Variable	Mean Difference Between ACP and Standard Care (95% CI)
Unadjusted: QALY gained from baseline	Total cost ($CAD)	−369 (−2,946 to 2,370)
	QALY	0.068 (−0.03 to 0.17)
	Mean ICER for ACP	−$5,465/QALY
	Probability of cost-effectiveness	
	WTP = $0	57.0%
	WTP = $50,000	88.6%
Adjusted: QALY gained from baseline	Total cost ($CAD)	−369 (−2,946 to 2,370)
	QALY gained	0.01 (−0.03 to 0.05)
	Mean ICER for ACP	−$3,4580/QALY
	Probability of cost-effectiveness	
	WTP = $0	43.3%
	WTP = $50,000	61.9%
Complete cases (follow-up for 1 y**)**	Total cost ($CAD)	−1,769 (−5,898 to 2,352)
	QALY	0.02 (−0.03 to 0.07)
	Mean ICER for ACP	−$82,393/QALY
	Probability of cost-effectiveness	
	WTP = $0	61.3%
	WTP = $50,000	74.9%
Inclusion of cost outlier (> $200,000)	Total cost ($CAD)	−2,982 (−9,045 to 1,905)
	QALY	0.01 (−0.03 to 0.05)
	Mean ICER for ACP	−$282,138/QALY
	Probability of cost-effectiveness	
	WTP = $0	60.7%
	WTP = $50,000	67.3%
Wait time ≤ 90 d	Total cost ($CAD)	−262 (−3,138 to 2,671)
	QALY	0.01 (−0.04 to 0.05)
	Mean ICER for ACP	−$29,369/QALY
	Probability of cost-effectiveness	
	WTP = $0	38.3%
	WTP = $50,000	58.1%
Wait time > 90 d	Total cost ($CAD)	−1,882 (−5,961 to 1,649)
	QALY	0.01 (−0.12 to 0.17)
	Mean ICER for ACP	−$271,531/QALY
	Probability of cost-effectiveness	
	WTP = $0	43.2%
	WTP = $50,000	68.5%
Non-PSG	Total cost ($CAD)	523 (−2,713 to 4,078)
	QALY	−0.02 (−0.08 to 0.05)
	Mean ICER for ACP	−$33,888/QALY
	Probability of cost-effectiveness	
	WTP = $0	11.2%
	WTP = $50,000	35.0%
PSG	Total cost ($CAD)	−582 (−4,286 to 3,137)
	QALY	0.03 (−0.02 to 0.09)
	Mean ICER for ACP	−$17,562/QALY
	Probability of cost-effectiveness	
	WTP = $0	54.9%
	WTP = $50,000	79.2%

ACP = alternative care provider; ICER = incremental cost-effectiveness ratio; PSG = polysomnography; QALY = quality-adjusted life-year; WTP = willingness to pay.

Among those with referral wait time ≤ 90 days, there was a mean cost saving and increase in mean QALY at 1 year in the ACP-led clinic, with a probability the ACP-led clinic was cost-effective at a WTP threshold of $50,000/QALY of 58.1%. Among those with referral wait time > 90 days, there was an even greater mean cost difference in favor of the ACP-led clinic compared with those with a wait time ≤ 90 days, resulting in a probability that the ACP-led clinic is cost-effective at a WTP threshold of $50,000/QALY of 68.5%.

## Discussion

In this 1-year follow-up study of an ACP-led clinic for patients with severe SDB, we demonstrated that PAP adherence was similar to physician-led care. There were sustained improvements in sleepiness scores, ODI, quality of life, and treatment satisfaction, with no statistically significant differences between groups. Economic analysis revealed slight improvements in health care-related costs and QALYs in the ACP-led clinic compared with the standard care group; however, this was not statistically significant. Overall, the ACP-led management strategy was likely to be cost-effective relative to standard care in this population of patients with severe SDB. These results were robust to sensitivity analysis and support the implementation of an ACP model of care to provide effective SDB care and reduce health care resource use for this population. To our knowledge, this is the first study to examine the cost-effectiveness of a model using ACPs in severe SDB management.

In the initial evaluation 3 months after treatment initiation, ACP management of SDB did not achieve noninferiority with respect to PAP adherence and was indeterminate. From 3 to 12 months, there was a reduction in PAP use of approximately 1 hour in the sleep physician group and 0.5 hours in the ACP group. Prior literature has demonstrated similar decreases in PAP adherence over time.^56^ It is possible the lesser reduction in adherence among patients from the ACP-led clinic reflects a sustained benefit of ACP care. However, PAP use at 3 months was 30 minutes lower in the ACP group compared with the standard care group, suggesting a floor effect in the ACP arm.^40^ Patient-reported outcomes were similar between groups at 1 year, compared with a greater improvement in Epworth Sleepiness Scale score reported in the ACP group at 3 months. These results may be due to significant attrition in study participants from 3 to 12 months or regression to the mean effects.

Prior studies have also demonstrated the potential economic benefits of newer models of OSA care. Antic et al^38^ demonstrated a model of care that incorporated specialist nurses and ambulatory testing was noninferior regarding improvements in sleepiness and over Australian dollar A$1,000 less costly than a traditional physician-led model that used polysomnography. Randomized trials of primary care management of uncomplicated OSA have demonstrated noninferiority and cost savings of approximately US $400.^57^, ^58^, ^59^, ^60^, ^61^ Management pathways using ambulatory testing have also been shown to be cost effective compared with laboratory polysomnography.^62^^,^^63^ The current study extends the findings of this prior research by demonstrating cost-effectiveness of ACP care in a more complex population with severe SDB.

The context of the current study is important to consider in interpreting the results and their generalizability to other settings. After the initial visit with a sleep physician or ACP based on randomization, there was significant overlap in follow-up care models which reflects the shared care approach at the FMC Sleep Centre. Although this comanagement model may have contributed to similar clinical and patient-reported outcomes, the economic evaluation demonstrates that these outcomes could be achieved more efficiently in the ACP arm from a health care system cost point of view. Physician-related costs were lower in the ACP arm and contributed to increased cost-effectiveness; we suspect this is due to lower costs of initial visits and also fewer physician follow-up visits as was observed in the 3-month analysis.^1^ Furthermore, ACPs in this study were respiratory therapists with several years of experience in SDB management. The ACPs did not undergo additional formal training in sleep disorders care before the study, but follow-up care was guided by established physician-approved protocols. Implementation of these results in other clinic settings must consider the training and scope of practice of health care providers functioning as ACPs.

The strengths of this study include the randomized design, comprehensive set of outcome measures, and a priori plan to capture concurrent resource use and quality of life alongside the clinical trial. The latter allowed us to assess cost-effectiveness of the ACP-led clinic relative to standard care from study enrollment to 12 months after treatment initiation, a longer time frame than many clinical trials in SDB. We also acknowledge important limitations of this study. First, this was a single-center study of patients with severe SDB and thus cannot be generalized to other patient types. Second, ongoing cost-effectiveness of ACP-led care compared with standard care beyond 12 months depends on long-term adherence, outcomes, and costs, which were not available at the time of analysis. Third, the sample size of the population was relatively small, and chosen to detect a difference in PAP adherence between the groups and was not powered to detect differences in health care costs. Despite this, our study reports relevant costs associated with both groups over a 1-year period. Additionally, we used methods such as bootstrapping and cost-effectiveness acceptability curves to characterize uncertainty in the estimates due to sample size limitations. Sensitivity analyses and subgroup analyses further add information about the robustness of the cost-effectiveness results. Importantly, the dropout rate at 12 months was significant and, although we do not suspect differential dropout between groups, may have led to selection bias. Finally, we focused on clinical and economic outcomes but did not include an evaluation of additional impacts of OSA treatment such as workplace productivity or injury. These additional evaluation domains could be explored in future studies.

## Interpretation

Overall, there was no statistically significant difference in clinical outcomes, mean costs, or quality of life among patients with suspected severe SDB in an ACP-led clinic vs standard management with a sleep physician. The probability the ACP-led clinic is cost-effective compared with standard care at a WTP threshold of $50,000/QALY was high, suggesting this model of care is a feasible delivery model with good value for money in this population. Further studies are needed to explore factors that may improve the cost-effectiveness of this model of care to optimize resource allocation decisions for this population.

## Funding/Support

The Canadian Sleep and Circadian Network which is supported by the Canadian Institutes of Health Research, and The Lung Association, Alberta and Northwest Territories.

## Financial/Nonfinancial Disclosures

The authors have reported to *CHEST* the following: E. D. P. reports research grants from provincial and national research funding bodies (CIHR, SHRF, RRC, and LungSask), AstraZeneca, Sanofi, GSK, and Saskatchewan Cancer Agency to her institution; received personal fees and honoraria for participation on advisory boards, lecture series, and educational events from AstraZeneca, Boehringer Ingelheim, COVIS Pharma, GSK, LungSask, Roche, Sanofi, and Valeo; and is president of the Canadian Thoracic Society, advisory board member for the Institute of Cancer Research for the Canadian Institutes for Health Research, and associate editor for *CHEST* (COPD section). S. R. P. reports honoraria from Jazz Pharmaceuticals for educational activities, unrelated to the submitted work; and grant funding from Jazz Pharmaceuticals, unrelated to the submitted work. None declared (A.-I. B., W. H. T., M. J. S., W. W. F., K. L. F.).

## Figures and Tables

**Supplementary Figure 2:**
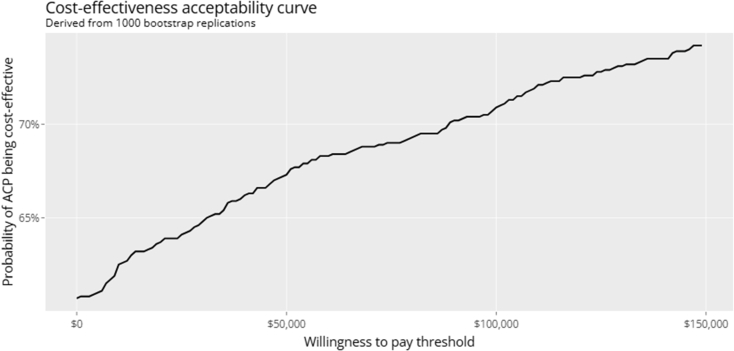
Cost-effectiveness acceptability curves for inclusion of cost outlier. Cost-effectiveness acceptability curve, derived from 1,000 bootstrap replications. Willingness to pay thresholds in Canadian dollars. ACP = alternative care provider.

**Supplementary Figure 3:**
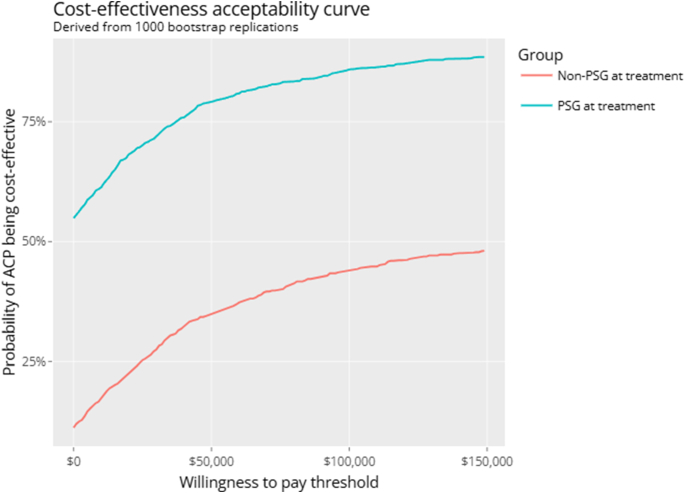


**Supplementary Figure 4:**
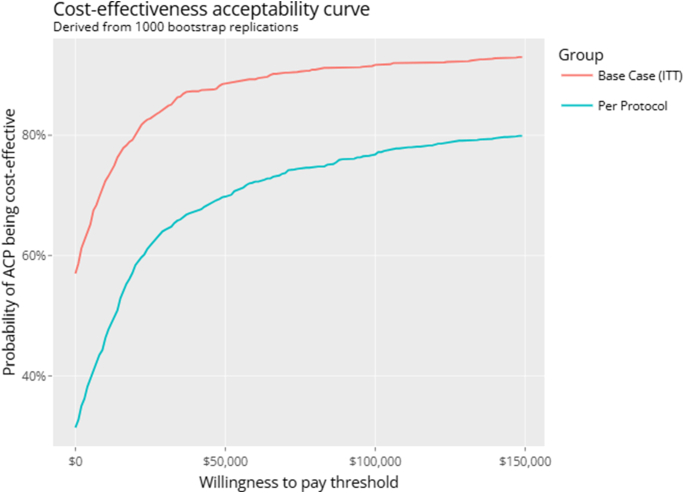


**Supplementary Figure 5:**
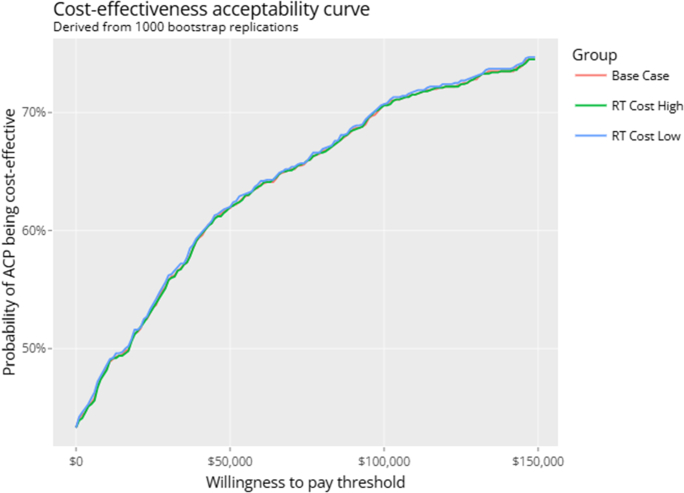


**Supplementary Figure 6:**
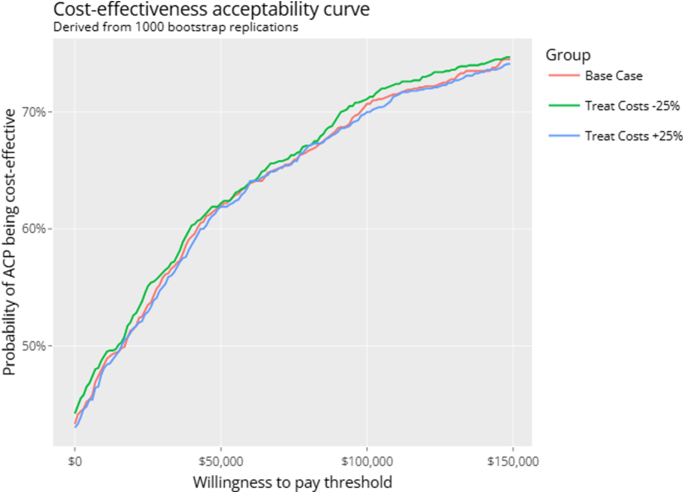


## Supplementary Data

e-Online Data

e-Online Data
