# Supplementary material for: Twelve-Month Follow-Up and Economic Evaluation of an Alternative Care Provider Clinic for Severe Sleep-Disordered Breathing
Source: Chest. 2026 Jan 24;169(6):1703–16. doi: 10.1016/j.chest.2026.01.008 (PMC13269686; doi:10.1016/j.chest.2026.01.008)
Supplement: e-Online Data [file mmc1.docx]

**e-FIGURE LEGENDS**

**Cost-Effectiveness Acceptability Curves for Sensitivity and Subgroup Analysis**

**e-Figure 1a – Unadjusted Cost-Effectiveness Acceptability Curves for Base Case (ITT) and Per Protocol Analyses.** Cost effectiveness acceptability curves derived from 1000 bootstrap replications. Willingness to pay thresholds in Canadian dollars. ACP = alternative care providers; ITT = intention to treat.

**e-Figure 1b – Cost-Effectiveness Acceptability Curves Adjusted for Treatment Cost Variation.** Cost-effectiveness acceptability curves derived from 1000 bootstrap replications. Treatment costs were adjusted by ±25%. Willingness to pay thresholds in Canadian dollars. ACP = alternative care providers.

**e-Figure 1c – Cost-Effectiveness Acceptability Curves Adjusted for RT Wage Variation.** Cost-effectiveness acceptability curves derived from 1000 bootstrap replications. RT salaries were adjusted from high to low cost. Willingness to pay thresholds in Canadian dollars. ACP = alternative care providers; RT = respiratory therapist.

**e-Figure 1d – Complete Case Cost-Effectiveness Acceptability Curve.** Derived from 1000 bootstrap replications. Included data from participants who had complete data collection over the 1-year study period. Willingness to pay thresholds in Canadian dollars. ACP = alternative care providers.

**2-Figure 1e – Cost-Effectiveness Acceptability Curve with Cost Outlier Inclusion**. Cost-effectiveness acceptability curve derived from 1000 bootstrap replications. Includes patient with healthcare costs exceeding $200,000. Willingness to pay thresholds in Canadian dollars. ACP = alternative care providers.

**e-Figure 1f – Cost-Effectiveness Acceptability Curves with Wait Time Variation.** Cost-effectiveness acceptability curves derived from 1000 bootstrap replications. Willingness to pay thresholds in Canadian dollars. ACP = alternative care providers.

**e-Figure 1g – Cost-Effectiveness Acceptability Curves for PSG and non-PSG use.** Cost-effectiveness acceptability curves derived from 1000 bootstrap replications. Non-PSG used HSAT alone. Willingness to pay thresholds in Canadian dollars. ACP = alternative care providers; HSAT = home sleep apnea testing; PSG = polysomnography.
